# Measuring vision loss beyond blindness: a severity transition in an ageing world

**DOI:** 10.1093/eurpub/ckag137

**Published:** 2026-07-22

**Authors:** Henry Bair

**Affiliations:** Wills Eye Hospital, Philadelphia, PA, United States

Over three decades, global eye health has recorded a paradox of progress. Among adults aged 50 years and older, the age-standardized prevalence of blindness fell substantially between 1990 and 2020, while moderate and severe vision impairment did not fall commensurately, and the absolute number of people living with impairment rose steeply [1]. The decline in blindness is rightly read as a public-health success. Yet a steep fall at the most severe end of the spectrum, alongside little movement at the moderate end, is the pattern expected under a particular kind of change. Legacy blindness-centred metrics, and even newer effective-coverage indicators, are not designed to show how many years of life are lived in each severity state. Interpreted through the framework that public health uses for such shifts, the pattern points to a severity transition: a redistribution of vision loss across severity states, in which blindness recedes while less severe impairment persists and grows in absolute numbers as populations age. Its consequences fall most heavily where eye-care systems are weakest.

Public health has long asked whether longer lives compress, expand or redistribute morbidity. Fries proposed that morbidity might be compressed into fewer late-life years if its onset were postponed faster than death [2]. A competing account holds that medical progress expands the years lived with disease. Manton described an intermediate possibility, dynamic equilibrium, in which impairment becomes more common as longevity rises even as its average severity falls [3]. Vision is a tractable test case, because its severity is clinically graded and its leading causes are treatable. Related health-expectancy methods have been applied to sensory impairment in cohort studies [4]. They have not yet been used for the global severity pattern that worldwide estimates now reveal.

That pattern is a divergence across severity. Among adults aged 50 years and older, age-standardized blindness prevalence fell by 28.5% between 1990 and 2020, moderate and severe vision impairment rose only slightly, by 2.5%, and mild impairment decreased slightly [1]. The two ends of the severity spectrum moved very differently. Blindness fell steeply, while moderate and severe impairment held nearly flat. Counts and rates must be read separately. Over the same period the number of people who were blind rose by 50.6% and the number with moderate and severe impairment by 91.7% [1]. These increases reflect population growth and ageing far more than any change in age-standardized prevalence. Cause-specific estimates sharpen the divergence. Among the avoidable causes that dominate this burden (cataract and undercorrected refractive error), age-standardized avoidable blindness fell while avoidable moderate and severe impairment did not, and the global reduction target set for these conditions was not met in an ageing population [5].

These trends make a simple verdict of compression or expansion incomplete. Pure compression does not fit, because the absolute number of people living with impairment has grown. Pure expansion does not fit either, because the prevalence of the most disabling state has contracted. The combined pattern is better understood as the redistribution of severity under demographic ageing that Manton termed dynamic equilibrium [3]. The composition of prevalent visual morbidity is shifting away from blindness and toward non-blinding impairment. The pattern is consistent with dynamic equilibrium without proving it, because repeated cross-sectional estimates cannot establish that any individual’s blindness has been averted into lesser impairment. A contributing mechanism is plausible. Vision impairment carries excess mortality that rises with severity [6]. Survival selection and treatment-mediated reductions in severity may both shape the population pattern, although reversibility varies substantially by cause and setting. Read this way, the severity transition is a plausible interpretation and a measurement hypothesis, to be tested rather than assumed.

The distinction matters in practice. Moderate and severe impairment is driven substantially by undercorrected refractive error and cataract, among the most cost-effectively treated conditions in medicine [5]. Yet this burden accrues disproportionately in low- and middle-income countries, where access is weakest, and it carries the downstream costs that make vision an ageing-systems concern: falls, reduced mobility, isolation, depression, cognitive decline, dependency and excess mortality [6, 7]. Global eye-health policy has already begun to move beyond blindness. The 2030 targets endorsed by the Seventy-fourth World Health Assembly, which seek a 40-percentage-point increase in effective refractive error coverage and a 30-percentage-point increase in effective cataract surgical coverage [8], embed a functional-acuity threshold rather than counting blindness alone. These indicators are necessary but incomplete for ageing societies. Prevalence and coverage capture how common each state is and how well services reach people at one point in time, yet they do not express how many years of life are lived in each severity state. A severity-specific measure would sit alongside blindness prevalence, moderate-and-severe impairment prevalence, and the effective refractive error and cataract surgical coverage targets rather than replace them, translating prevalence and mortality into the expected years lived in each vision state.

Both questions, which pattern holds and how many years are lived in each state, need the same instrument. Health expectancy supplies it, partitioning remaining life into years lived in defined health states, in the tradition Sullivan established [9]. Vision has entered that literature only partially. Studies have estimated sensory life expectancies using single impairment thresholds [4], but none provides a routinely reported, global measure decomposed by visual-acuity severity. The measure proposed here, a severity-specific distance-vision health expectancy, would estimate how many of the remaining years at a given age are lived with normal sight and how many with mild, moderate-and-severe, or blind vision, and would track those years over time. It is buildable now by the Sullivan method, combining age-specific prevalence in each severity state with national life tables, from the presenting-acuity data that surveys such as the Rapid Assessment of Avoidable Blindness already collect (Box 1) [10]. It should rest on presenting vision, which reflects the impairment people actually live with and matches how global estimates and the coverage targets are framed; best-corrected vision, recorded alongside, would separate undercorrected refractive error from other causes. Feasibility would depend on the quality and frequency of surveys, the comparability of visual-acuity definitions, and coverage of countries with weaker eye-health information systems, which argues for building the measure where data already allow and extending it as surveys improve.

Box 1. A severity-specific distance-vision health expectancy: a proposed complementary metric for global eye-health monitoring

**What it measures:** the expected remaining years that a population at a given age will live in each vision state (normal vision, mild impairment, moderate-and-severe impairment, and blindness), under current mortality and prevalence conditions.

**How it is built:** the Sullivan method combines age-specific prevalence by severity with national life tables. The prevalence inputs are already gathered by population-based eye surveys, including the Rapid Assessment of Avoidable Blindness, and estimates can be reported by age, sex, region and World Bank income group.

**What it answers that coverage and prevalence cannot:** whether added years of life are spent with better or worse vision; whether rising effective coverage is accompanied by fewer years lived blind, fewer years lived with moderate-and-severe impairment, or both; and whether countries with comparable coverage differ in years lived with functional sight.

**What it would inform:** the scale and location of cataract and refractive services, rehabilitation and low-vision capacity, and workforce and social-care planning for the non-blinding impairment that current targets do not capture.

Used for monitoring, the measure would test more directly than prevalence whether population years lived blind are falling while years lived with lesser impairment rise. Its primary purpose would be population-level monitoring, both globally and between countries, with national planning of cataract, refractive, rehabilitation and low-vision services, and equity comparison across settings, as secondary applications. Because it can be stratified by age, sex, region and income group, it would show where people spend the most years with avoidable impairment, and would separate national trajectories that global averages combine: settings where blindness is falling while non-blinding impairment rises, and settings where persistent blindness coexists with growing moderate impairment. The measure applies equally to high-income ageing societies, including across Europe, where systems increasingly monitor healthy longevity and long-term-care demand and where non-blinding impairment bears on falls, frailty, autonomy and social-care need.

Falling blindness is real progress, but it has changed the kind of vision loss that ageing populations carry without eliminating it. Naming that change as a severity transition, and measuring it, is the first step toward resourcing the years of impaired vision now being lived, above all in the settings that bear the largest, most treatable and least-measured share.

Conflict of interest: None declared.

## Data Availability

No new data were generated or analysed in support of this viewpoint; all data discussed are available in the cited published sources.
